# Localization and Maintenance of Engrafted Mesenchymal Stem Cells Administered via Renal Artery in Kidneys with Ischemia-Reperfusion Injury

**DOI:** 10.3390/ijms22084178

**Published:** 2021-04-17

**Authors:** Yumi Yamada, Ayumu Nakashima, Shigehiro Doi, Naoki Ishiuchi, Ryo Kanai, Kisho Miyasako, Takao Masaki

**Affiliations:** 1 Department of Nephrology, Hiroshima University Hospital, 1-2-3 Kasumi, Minami-ku, Hiroshima 734-8551, Japan; yumiyamada@hiroshima-u.ac.jp (Y.Y.); sdoi@hiroshima-u.ac.jp (S.D.); ishiuchi@hiroshima-u.ac.jp (N.I.); d163551@hiroshima-u.ac.jp (R.K.); d192251@hiroshima-u.ac.jp (K.M.); 2 Department of Stem Cell Biology and Medicine, Graduate School of Biomedical & Health Sciences, Hiroshima University, 1-2-3 Kasumi, Minami-ku, Hiroshima 734-8553, Japan

**Keywords:** ischemia-reperfusion injury, acute kidney injury, mesenchymal stem cell, renal artery, renal fibrosis

## Abstract

Mesenchymal stem cells (MSCs) are a potential therapeutic tool for preventing the progression of acute kidney injury (AKI) to chronic kidney disease (CKD). Herein, we investigated the localization and maintenance of engrafted human bone marrow-derived MSCs in rats subjected to a renal ischemia-reperfusion injury (IRI) and compared the effectiveness of two intravascular injection routes via the renal artery or inferior vena cava. Renal artery injection of MSCs was more effective than intravenous injection at reducing IRI-induced renal fibrosis. Additionally, MSCs injected through the renal artery persisted in injured kidneys for over 21 days, whereas MSCs injected through the inferior vena cava survived for less than 7 days. This difference may be attributed to the antifibrotic effects of MSCs. Interestingly, MSCs injected through the renal artery were localized primarily in glomeruli until day 3 post-IRI, and they decreased in number thereafter. In contrast, the number of MSCs localized in tubular walls, and the interstitium increased gradually until day 21 post-IRI. This localization change may be related to areas of damage caused by IRI because ischemia-induced AKI leads to tubular cell damage. Taken together, these findings suggest renal artery injection of MSCs may be useful for preventing the progression of AKI to CKD.

## 1. Introduction

Patients recovering from acute kidney injury (AKI) have a high risk of progression to chronic kidney disease (CKD), end-stage kidney disease, and death [1]. For example, older individuals [2,3], diabetic patients [4], and postoperative patients [5] experience decreased recovery of kidney function from AKI and increased risk of progression to advanced-stage CKD. AKI has various causes, which complicates its management, and there are currently no standard therapies for its treatment. Therefore, establishing a novel therapy for preventing progression from AKI to CKD is an important issue that warrants researchers’ attention.

Mesenchymal stem cells (MSCs) have emerged as an advanced tool for tissue-regenerative therapy because of their paracrine effects and differentiative potential [6]. Additionally, MSCs can suppress inflammation and oxidative stress responses [7]. MSCs have been shown to ameliorate renal dysfunction and tissue injury caused by toxin- and cisplatin-induced experimental AKI [8], ischemia-reperfusion injury (IRI) [9,10,11], and sepsis [12]. Therefore, using MSCs to treat AKI is a novel approach that is currently of interest. However, there is no established route for delivering MSCs to injured kidneys. It is, therefore, important to analyze differences in localization and periods of engraftment of MSCs in injured kidneys that result from different routes of administration.

Using a rat model of ureteral obstruction, Asanuma et al. investigated differences in the amount of time engrafted MSCs were detectable in kidneys following different administration routes. According to their results, MSCs injected through the renal artery were detectable in injured kidneys for up to 4 weeks, whereas intravenously injected MSCs were mostly present in the lung with few in the kidneys [13]. In addition, the previous study showed that hMSCs administered through the renal artery could maintain renal function for 24 h post-IRI [11]. These observations suggest that under conditions of AKI, MSCs injected through the renal artery may be more effective than those injected intravenously because of long-term engraftment of MSCs in injured kidneys. However, at present, the antifibrotic effects and long-term engraftment of MSCs delivered via different administration routes in models of AKI remain unclear. In the present study, we investigated the time course of localized distribution of MSCs and their therapeutic effects in a rat model of IRI.

## 2. Results

### 2.1. Injection of hMSCs into IRI Kidney

**Figure 1:**
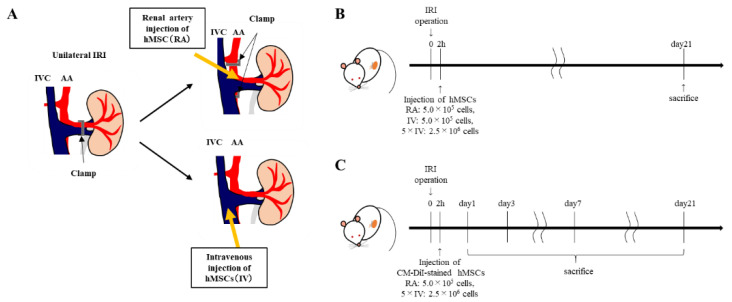
Ischemia-reperfusion injury (IRI) experimental protocol. (**A**) After the left kidney was visualized, the left renal artery was clamped for 60 min using atraumatic vascular clamps to establish the model of IRI. Sixty minutes after reperfusion, rats were injected through the renal artery or inferior vena cava. (**B**) After IRI, rats in the renal artery (RA) group were injected with 5 × 10^5^ hMSCs diluted in 0.2 mL PBS through the renal artery, while rats in the IV group were injected with the same amount of cells via the inferior vena cava. Rats in the 5 × IV group were intravenously injected with 2.5 × 10^6^ hMSCs diluted in 0.3 mL PBS. To evaluate the antifibrotic effect of hMSCs, rats were sacrificed on day 21. (**C**) To determine the engraftment period and localization of hMSCs in IRI kidneys, rats in the RA group, received an intra-arterial injection of 5 × 10^5^ CM-DiI-stained hMSCs diluted in 0.2 mL PBS, while rats in the 5 × IV group received 2.5 × 10^6^ CM-DiI-stained hMSCs intravenously. Rats were sacrificed on days 1, 3, 7, 21, and 42 post-IRI. Abbreviations: IRI, ischemia-reperfusion injury; hMSCs, human mesenchymal stem cells; RA, renal artery injection; IV, intravenous injection.

We induced IRI by clamping the left renal artery. Sixty minutes after reperfusion, rats were injected through the renal artery or inferior vena cava (Figure 1A). Rats in the RA group were injected with 5 × 10^5^ hMSCs/rat diluted in 0.2 mL PBS through the renal artery, while the IV group was injected with the same amounts of MSCs via the inferior vena cava. Rats in the 5 × IV group were injected with 2.5 × 10^6^ hMSCs diluted in 0.3 mL PBS were injected through the inferior vena cava (Figure 1B). To evaluate the antifibrotic effect of hMSCs, rats were sacrificed on day 21. Next, to track the localization of hMSCs in vivo, cells were stained with chloromethylbenzamido (CM)-DiI before administration. Stained hMSCs were injected through the renal artery following IRI. Rats were sacrificed on days 1, 3, 7, 21, and 42 post-IRI (Figure 1C).

### 2.2. Antifibrotic Effects of hMSCs in IRI Kidneys: Comparison between Renal Arterial and Intravenous Injection

**Figure 2:**
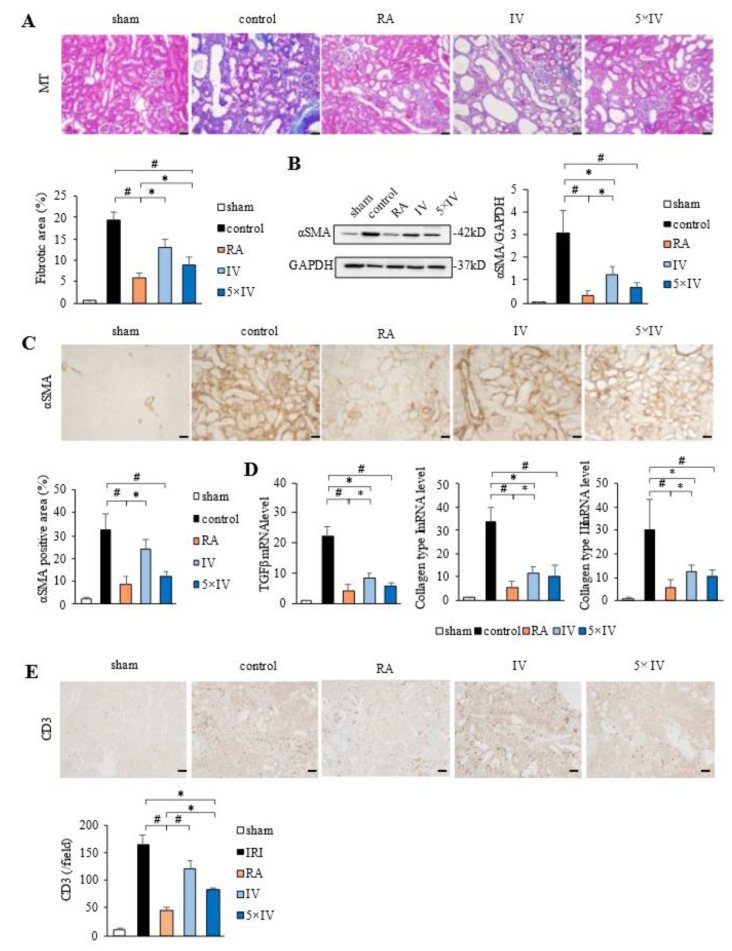
Therapeutic effects of hMSCs in the post-IRI kidney. (**A**) Masson’s trichrome staining showed the antifibrotic effects of hMSCs and renal morphology at 21 days post-IRI. Graphs show the fibrotic area determined using Masson’s trichrome staining (×100, scale bar = 50 µm). (**B**) Western blot analysis of α-SMA in kidney cortex at 21 days post-IRI. The graph shows the quantification of α-SMA levels normalized to GAPDH. (**C**) Immunostaining of α-SMA shows the antifibrotic effects of hMSCs and renal morphology after IRI. Representative photos show the fibrotic area determined by immunostaining of α-SMA (×100, scale bar = 50 µm) (**D**) Graphs showing the mRNA levels of TGF-β, collagen type I, and collagen type III at day 21 post-IRI. (**E**) Representative images of immunostaining of CD3 at day 21 post-IRI (×100, scale bar = 50 µm). Graphs show quantification of CD3-positive cells. # *p* < 0.01, * *p* < 0.05. Abbreviations: hMSCs, human mesenchymal stem cells; IRI, ischemia-reperfusion injury; RA, renal artery injection; IV, intravenous injection.

To evaluate the antifibrotic effects of hMSCs, we first performed MT staining of kidney sections at 21 days post-IRI. As shown in Figure 2A, IRI induced severe fibrosis in the control group, while intravenous injection of hMSCs (IV group) suppressed the fibrotic area. Moreover, in rats injected with hMSCs via the renal artery (RA group), the reduction in the fibrotic area was more significant than that of the IV group. Intravenous injection of quintuple the amount of hMSCs (5 × IV group) also caused a reduction of the fibrotic area, although it was significantly lower than that observed in the RA group. Similarly, compared with the control group, levels of α-SMA protein (a marker of myofibroblast differentiation) induced by IRI were suppressed by intravenous injection of hMSCs (IV group) (Figure 2B). The reduction was more significant in the RA group than the IV group. Intravenous injection of quintuple the amount of hMSCs (5 × IV group) also suppressed α-SMA protein levels, although the change was not statistically significant compared with that in the RA group. Immunostaining also revealed the α-SMA-positive area was significantly reduced in the RA group compared with the IV group (Figure 2C). Compared with the IV group, the α-SMA-positive area was more reduced in the 5 × IV group, although it did not reach statistical significance as with the RA group. Furthermore, mRNA levels of TGF-β (a profibrotic marker) and collagen types I and III (extracellular matrix protein markers) were increased in the control group, while an infusion of hMSCs suppressed these increases. No significant difference was found in collagen type I mRNA levels among the three groups. Regarding TGF-β and collagen type III, suppression was stronger in the RA group than the IV group, although no significant difference was found between the RA group and the 5 × IV group (Figure 2D). To evaluate the anti-inflammatory effect of hMSCs, we examined the expression of CD3 (a T cell marker). Immunohistochemical staining revealed that the accumulation of CD3-positive cells increased in the IRI group. The injection of hMSCs suppressed the infiltration of these cells in the IV and 5 × IV groups. The suppressive effect was most significant in the RA group (Figure 2E).

### 2.3. Comparison of Localization between Arterially and Venously Delivered hMSCs

**Figure 3:**
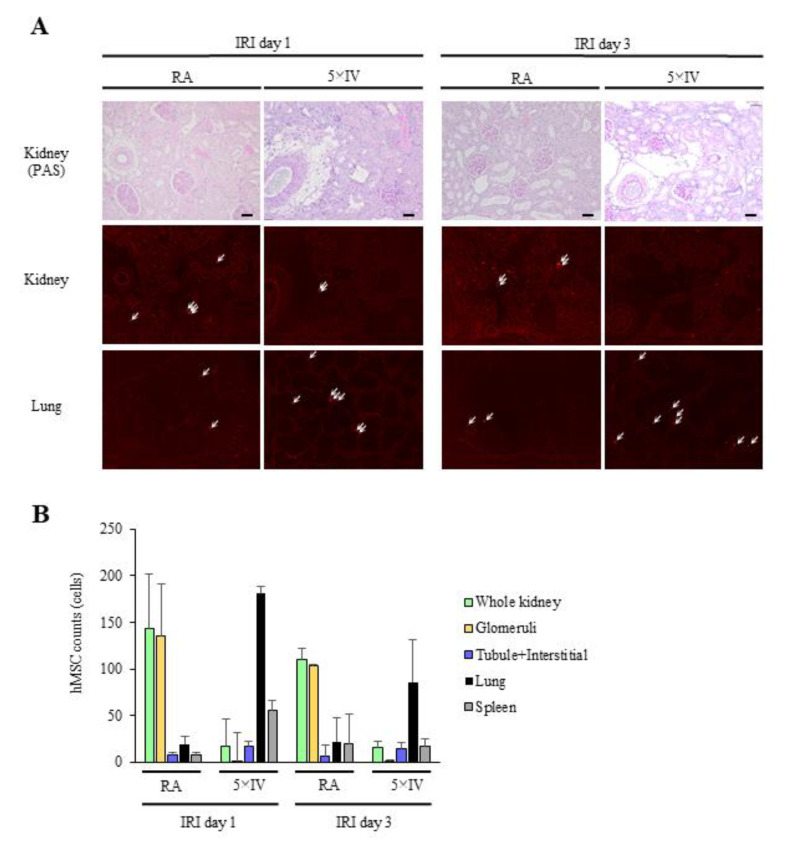
Examination of post-IRI kidney sections comparing the different routes of administration of hMSCs. (**A**) Representative distribution of arterially delivered CM-DiI-stained hMSCs (RA group, 5 × 10^5^ hMSCs/rat, red-labeled cells; arrows) to the IRI kidney shows that significantly more implanted hMSCs were retained compared with the intravenously injected group (5 × IV group, 2.5 × 10^6^ hMSCs/rat, red-labeled cells; arrows). The photos of PAS staining above show the same kidney sections, ×100. (scale bar = 50 µm) (**B**) Difference between the number of CM-DiI-stained hMSCs delivered intra-arterially and intravenously in 10 random fields (×100) in kidney, lung, and spleen at days 1 and 3 post-IRI. In the intravenously injected group, grafted hMSCs accumulated primarily in the lung. Abbreviations: hMSCs, human mesenchymal stem cells; IRI, ischemia-reperfusion injury; RA, renal artery injection; IV, intravenous injection; PAS, periodic acid-Schiff.

Next, we investigated differences in engraftment period and localization of hMSCs in IRI kidneys following renal arterial or intravenous injection of CM-DiI-stained hMSCs. In the RA group, most CM-DiI-stained cells detected in kidney sections were localized in glomeruli on days 1 and 3 post-IRI (Figure 3A). Additionally, significantly fewer stained cells were detected in the lung and spleen compared with the kidney. Conversely, even when quintuple the amount of hMSCs was injected (5 × IV group), compared with the RA group, only a small number of stained cells were detected in kidney sections at days 1 and 3 post-IRI, and they were primarily localized in tubular walls and the interstitium (Figure 3A,B). In the 5 × IV group, stained cells were localized more prominently in the lung compared with the kidney. Regarding the spleen, the number of stained cells observed on day 1 post-IRI was higher in the 5 × IV group than the RA group, although no difference was found between the two groups on day 3. In the 5 × IV group, stained cells were undetected in the kidney on day 7 (data are not shown).

### 2.4. Localization of Arterially Delivered hMSCs

**Figure 4:**
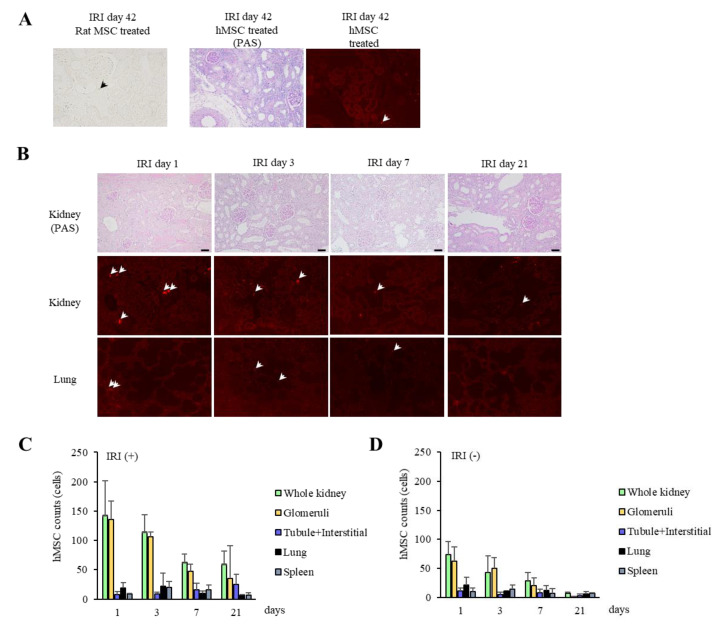
Time course of engraftment of hMSCs after IRI. (**A**) Both rat MSCs (black arrow) and hMSCs (white arrows) were sparsely observed at day 42 post-IRI. (**B**) Localization of arterial delivered CM-DiI-stained hMSCs (5 × 10^5^ hMSCs/rat, red-labeled cells; arrows) in kidney and lung tissue sections. PAS stain photos clarify the structure of kidney sections with images showing CM-DiI-stained hMSCs (middle). CM-DiI-stained hMSCs appeared mostly in glomeruli at day 1 post-IRI and were gradually detected in tubular walls and the interstitium (×100, scale bar = 50 µm). (**C**,**D**) The number of CM-DiI-stained hMSCs delivered arterially in 10 random fields (×100) in kidney, lung, and spleen at days 1, 3, 7, and 21 post-IRI, and without undergoing IRI. When hMSCs were injected intra-arterially, most engrafted hMSCs were found in the kidney. Abbreviations: hMSCs, human mesenchymal stem cells; IRI, ischemia-reperfusion injury; PAS, periodic acid-Schiff.

We recently reported that MSCs from EGFP-positive rats persisted for 21 days post-IRI [14]. We further traced MSCs from EGFP-positive rats in IRI kidneys and found they were sparsely detected on day 42 post-IRI (Figure 4A). Similarly, we examined the engraftment of CM-DiI-stained hMSCs in the kidneys at day 42 post-IRI. Under fluorescence microscopy, stained cells were sparsely detected at day 42 (Figure 4A). Therefore, we investigated the localization and number of CM-DiI-stained hMSCs engrafted over time (up to 21 days) in IRI kidneys (Figure 4B). Although the number of stained cells observed in whole kidneys was highest on day 1 post-IRI and decreased over time (*p* for trend = 0.01), the localization of stained cells changed from glomeruli to tubular walls and the interstitium (Figure 4B,C and Supplementary Materials Table S1A). Stained cells were predominantly localized in glomeruli on days 1 and 3, whereas detecting these cells in tubular walls and the interstitium increased gradually from day 7 to day 21. Subsequently, we investigated the engraftment period and localization of renal artery-injected hMSCs in kidneys not subjected to IRI. Although stained cells were detected in kidney, lung, and spleen sections, the number of cells in kidneys not subjected to IRI was much lower than that in IRI kidneys, and they were almost absent at day 21 (Figure 4D and Supplementary Materials Table S1B). No stained cells were detected in kidneys not subjected to IRI (i.e., sham-operated kidneys) or IRI kidneys without arterial injection of hMSCs.

## 3. Discussion

This study showed that MSCs injected through the renal artery exhibited stronger antifibrotic effects than those injected intravenously in a rat model of IRI. Furthermore, MSCs injected through the inferior vena cava became mostly confined in the lung and were sparsely observed in post-IRI kidneys, whereas MSCs injected through the renal artery were clearly detected in injured kidneys until day 21 post-IRI. Interestingly, MSCs injected through the renal artery were localized primarily in glomeruli until day 3 post-IRI and were localized in tubular walls and the interstitium at day 21 post-IRI. This localization change may be attributed to areas of damage induced by IRI because ischemia-induced AKI leads to tubular cell damage [9,15].

Our results indicate that when infusing the same amount of hMSCs to IRI kidneys, renal arterial injection is more suitable than intravenous injection for hMSCs to exert their antifibrotic effects. Moreover, delivering quintuple the amount of hMSCs through the inferior vena cava was not as effective as an injection through the renal artery. Cai et al. compared the effect of rat bone-marrow-derived MSCs on serum creatinine level after 24 h in a rat model of IRI following three different administration routes (tail vein, carotid artery, and renal artery). They showed that MSCs administered through the renal artery could maintain renal function for 24 h post-IRI [11]. Consistent with this report. We demonstrated that MSCs injected through the renal artery exerted stronger antifibrotic effects than those injected intravenously, at 21 days post-IRI. In contrast, the weak renal antifibrotic effects from an intravenous injection of MSCs may have been because of insufficient retention of MSCs in the kidney. With the intravenous injection, numerous cells are lost in the systemic circulation, especially in the lungs [16]. Indeed, when we performed intravenous injections, small numbers of MSCs were detected in the kidney. Compared with the systemic route, injection through the renal artery may deliver a larger number of MSCs to the ischemic area and significantly increase homing efficiency to the kidney. Intra-arterial delivery of MSCs is also gaining interest for the treatment of other diseases, such as brain stroke [17]. Our results suggest that injection of MSCs through the main artery of an organ is the most effective method for achieving MSC-induced maintenance of the organ’s function.

We also assessed the number of engrafted hMSCs over time and their localization in IRI kidneys. Larger numbers of hMSCs were detected in IRI kidneys following injection through the renal artery. Engrafted hMSCs injected through the renal artery were clearly detected in IRI kidneys for up to 21 days and were sparsely detected by 42 days post-IRI. Conversely, following intravenous injection, hMSCs were mostly observed in the lung, with a small number observed in IRI kidneys. Furthermore, they disappeared by day 7 post-IRI. Our results indicate that in the post-IRI kidney, long-term engraftment following delivery by renal artery injection may enhance the antifibrotic effects of hMSCs. Direct delivery of MSCs to the injured kidney may prevent cell washout and retention because it can circumvent cell trapping in other filtering organs. Interestingly, the localization of hMSCs in injured kidneys changed over time. They were predominantly detected in glomeruli until day 3 post-IRI and decreased in number thereafter. In contrast, the number of hMSCs localized in tubular walls and the interstitium increased gradually throughout the observation period. AKI because renal IRI induces tubular cell disruption resulting in renal injury [14,18]. Because MSCs migrate to injury sites [19,20], MSCs injected through the renal artery may first become trapped in glomeruli, then slowly migrate to the lesions induced by IRI. The existence of fewer hMSCs in kidneys not subjected to IRI may support this notion.

MSC administration can ameliorate renal injury; however, cell therapy’s delivery routes vary in animal models [10,11,21] and in human clinical trials [22,23,24]. We demonstrated two intravascular routes for delivering hMSCs for the treatment of IRI kidneys. Direct intra-arterial delivery of hMSCs, without leakage to other organs, is therapeutically effective with fewer hMSCs. Furthermore, according to our results, over quintuple, the number of hMSCs delivered by intravenous injection was required to achieve roughly the same therapeutic effect conferred with renal artery injection. Compared with renal artery injection, intravenous injection is much simpler for administering MSCs. However, in the clinical setting, the preparation of a sufficient number of MSCs for treatment may be challenging. Additionally, intravenously injected MSCs tend to get trapped in the lung [13,25], which, as previously reported, is a risk for developing pulmonary thromboembolism [26,27]. Moreover, an excessive amount of MSCs also increases the risk of tissue injury associated with occlusion and embolization [11,24]. Therefore, further studies are required to identify an adequate number of MSCs for injection through the renal artery and to design appropriate clinical trials.

## 4. Materials and Methods

### 4.1. Rats

Eight-week-old male Sprague-Dawley (SD) rats (weight: 270–330 g) were from Charles River Laboratories, Yokohama, Japan. Six-week-old male CAG-enhanced green fluorescent protein-transgenic (CAG-EGFP-transgenic) SD rats (Japan SLC, Shizuoka, Japan) were used to harvest bone marrow.

### 4.2. Study Approval

All experimental procedures involving animals were performed according to the “Guide for the Care and Use of Laboratory Animals, eighth edition, 2010” (National Institutes of Health, Bethesda, MD, USA), and approved by the Institutional Animal Care and Use Committee of Hiroshima University (Hiroshima, Japan) (Permit Number: A19-47).

### 4.3. Preparation of Rat and Human MSCs

Rat MSCs were harvested from the bone marrow of CAG-EGFP-transgenic SD rats as previously described [28]. Bone marrow-derived human MSCs (hMSCs) were from Riken, Japan. We reported that culturing MSCs in a serum-free medium enhances their anti-inflammatory and antifibrotic capacities [28]. Therefore, in this study, we cultured MSCs in serum-free STK medium (DS Pharma Biomedical, Osaka, Japan) at 37 °C in humidified air containing 5% CO_2_. Cells were passaged after reaching 80–90% confluence. For all experiments, we used rat MSCs collected at passage five and hMSCs collected at passages five to six.

### 4.4. Rat Model of Renal IRI

Before inducing IRI, rats were anesthetized with intraperitoneal administration of a cocktail of three anesthetic agents (0.15 mg/kg medetomidine, 2.0 mg/kg midazolam, and 2.5 mg/kg butorphanol). After opening the abdomen, the left kidney and left renal artery were visualized. Next, the left renal artery was clamped for 60 min using atraumatic vascular clamps on a heating blanket (rats in the sham-operated group were not clamped). We visually confirmed kidney reperfusion after clamps were removed. Sixty minutes after reperfusion, hMSCs (5 × 10^5^ cells/rat) diluted in 0.2 mL PBS were injected through the renal artery with the abdominal aorta clamped above and below the left renal artery bifurcation (RA group, *n* = 5) or through the inferior vena cava (IV group, *n* = 5). Rats in the 5 × IV group (injected with 2.5 × 10^6^ hMSCs diluted in 0.3 mL PBS, *n* = 5) and control group (injected with 0.2 mL PBS, *n* = 5) were injected via the inferior vena cava. Because renal artery injection can cause severe bleeding, an injection of MSCs was performed using an extra-fine needle (a 32-gauge needle with a wide inner diameter) (React System, Osaka, Japan). Sham-operated rats (sham group, *n* = 5) underwent a midline abdominal incision without clamping of the left renal artery. To assess the effects of hMSC administration, rats from the different groups were euthanized on day 21 post-IRI using the mixture of anesthetic agents described above. Rat kidneys were quickly isolated and sectioned. Some were fixed in 10% formaldehyde for histological analysis, while the remaining sections were stored at −80 °C for further studies.

### 4.5. CM-DiI Staining of hMSCs

To track the fate of hMSCs in vivo, hMSCs were stained with chloromethylbenzamido (CM)-DiI (CellTrackerTM CM-DiI, C7000, Thermo Fisher Scientific, Waltham, MA, USA) according to the manufacturer’s instructions. Staining efficacy was >98%. Stained hMSCs were injected via the renal artery following IRI as described above. As shown in Figure 1C, rats were sacrificed on days 1, 3, 7, 21, and 42 post-IRI. Kidney, lung, and spleen samples were harvested, fixed in 10% formaldehyde, and processed in paraffin. Stained hMSCs were also injected via the inferior vena cava following IRI, and samples were collected on days 1 and 3, fixed in 10% formaldehyde, and processed in paraffin. Harvested tissue samples were sectioned (4-µm), and staining was detected using a fluorescence microscope (KEYENCE, Osaka, Japan). The number of CM-DiI-stained hMSCs was counted in 10 random fields (×200, *n* = 5).

### 4.6. Western Blot

Preparation of kidney samples for Western blot was performed as previously described [29]. Blots were incubated overnight with an antibody against alpha-smooth muscle actin (α-SMA) (1:1000; Clone 1A4, Cat# A2547, Sigma-Aldrich, Saint Louis, MO, USA). To quantify bands, we used ImageJ software (version 1.47v; National Institutes of Health) and normalized data to levels of glyceraldehyde 3-phosphate dehydrogenase (GAPDH, Cat# G8795, Sigma-Aldrich, Saint Louis, MO, USA).

### 4.7. Histopathology

Rat kidneys were fixed in 10% formaldehyde, embedded in paraffin, cut into 2 μm sections, and stained with Masson’s trichrome (MT) or periodic acid-Schiff (PAS) reagents for examination under light microscopy. MT staining was performed as previously described [28,29]. Areas of interstitial fibrosis were viewed at ×100 magnification and analyzed using Lumina Vision (MITANI Corporation, Fukui, Japan). PAS staining was performed according to the manufacturer’s instructions.

### 4.8. Immunohistochemistry

Immunohistochemistry was performed as previously described [29]. We stained 4 μm thick sections from kidneys harvested at day 21, with mouse monoclonal anti-α-SMA antibody (A-2547; Sigma-Aldrich, Saint Louis, MO, USA) and rabbit polyclonal anti-CD3 antibody (IR503; Dako, Santa Clara, CA, USA). Reaction products were detected using the avidin-biotin-peroxidase complex method (Vector Laboratories, Burlingame, CA, USA), and color reactions were developed in 3,3-diaminobenzidine (Sigma-Aldrich, Saint Louis, MO, USA) and hydrogen peroxide. Five non-overlapping fields of the renal cortex were selected (×100, *n* = 5), and images were qualitatively analyzed using ImageJ software.

### 4.9. Quantitative Real-Time Reverse Transcription–Polymerase Chain Reaction

RNA extraction and quantitative real-time reverse transcription–polymerase chain reaction were performed as previously described [30]. Primers and TaqMan probes (TaqMan Gene Expression Assay) were from Applied Biosystems (Foster City, CA, USA). The assay identification codes for each gene were as follows: TGF-β (Rn00572010_m1), collagen type I (Rn01463848_m1), and collagen type III (Rn01437681_m1). mRNA levels were normalized to the level of 18S rRNA.

### 4.10. Statistical Analysis

Data are presented as mean ± standard deviation. Differences among groups were analyzed using Kruskal–Wallis test with Dunn post-test. Differences between the two groups were compared using Mann–Whitney test. Statistical significance was set at *p* < 0.05.

## 5. Conclusions

This study showed that direct delivery of hMSCs through the renal artery was the most effective method for reducing IRI-induced renal fibrosis, thereby circumventing cell trapping in other organs. Our results may support clinical trials for the use of hMSCs to alleviate kidney injury.

## Data Availability

Datasets analyzed during the present study are available from the corresponding author upon reasonable request.

## Supplementary Materials

The following are available online at https://www.mdpi.com/article/10.3390/ijms22084178/s1.

## Abbreviations

AKI: acute kidney injury; ANOVA: analysis of variance, CKD: chronic kidney disease; IRI: ischemia-reperfusion injury; MSCs: mesenchymal stem cells; MT: Masson’s trichrome; PAS: periodic acid-Schiff; SD: Sprague-Dawley; SMA: smooth muscle actin; TGF: transforming growth factor.
