# Supplementary material for: Localization and Maintenance of Engrafted Mesenchymal Stem Cells Administered via Renal Artery in Kidneys with Ischemia-Reperfusion Injury
Source: Int J Mol Sci. 2021 Apr 17;22(8):4178. doi: 10.3390/ijms22084178 (PMC8072868; doi:10.3390/ijms22084178)

Supplementary files

Localization and maintenance of engrafted mesenchymal stem cells administered via renal artery in kidneys with ischemia-reperfusion injury

Yumi Yamada[1], Ayumu Nakashima[1,2], Shigehiro Doi[1], Naoki Ishiuchi[1], Ryo Kanai[1], Kisho Miyasako[1] and Takao Masaki[1]

[1]Department of Nephrology, Hiroshima University Hospital, 1-2-3 Kasumi, Minami-ku, Hiroshima, Hiroshima 734-8551, Japan

[2]Department of Stem Cell Biology and Medicine, Graduate School of Biomedical & Health Sciences, Hiroshima University, 1-2-3 Kasumi, Minami-ku, Hiroshima, Hiroshima 734-8553, Japan

This file includes supplementary table 1 and western blotting uncropped gel images.

Supplementary table file.

Supplemental table 1. Time course of engraftment of hMSCs. The number of CM-DiI-stained hMSCs delivered arterially in 10 random fields (×100) in kidney, lung, and spleen at days 1, 3, 7, and 21 post-IRI (A), and without undergoing IRI (B).

A

hMSC counts (cells), IRI(+)				
	day 1 (n=5)	day 3 (n=5)	day 7 (n=5)	day 21 (n=5)
Whole kidney	143.2 ± 58.1	114.6 ± 29.6	62.7 ± 13.6	59.4 ± 22.9
Glomeruli	135.6 ± 55.8	106.2 ± 31.5	46.8 ± 8.3	34.8 ± 12.2
Tubule+Interstitial	7.6 ± 3.0	8.4 ± 5.5	15.8 ± 11.5	24.6 ± 17.5
Lung	19 ± 8.7	22 ± 8.2	9.2 ± 4.5	6.6 ± 1.4
Spleen	8.2 ± 1.7	19.4 ± 10.4	16.2 ± 7.9	6.4 ± 4.5

B

hMSC counts (cells), IRI(-)				
	day 1 (n=5)	day 3 (n=5)	day 7 (n=5)	day 21 (n=5)
Whole kidney	73.5 ± 22.7	42.8 ± 28.5	28.5 ± 14.7	7.0 ± 2.1
Glomeruli	62.0 ± 24.8	50.0 ± 18.5	20.3 ± 14.0	0.8 ± 1.6
Tubule+Interstitial	11.5 ± 5.7	5.25 ± 3.6	8.3 ± 5.1	6.2 ± 2.9
Lung	21.0 ± 14.0	11 ± 0.8	12.5 ± 7.9	7.6 ± 4.0
Spleen	10.5 ± 5.9	19.7 ± 7.3	7.5 ± 7.6	7.6 ± 0.8

Data are presented as the mean ± SD.
hMSCs, human mesenchymal stem cells; IRI, ischemia-reperfusion injury

Western blotting uncropped gel images

Figure 2

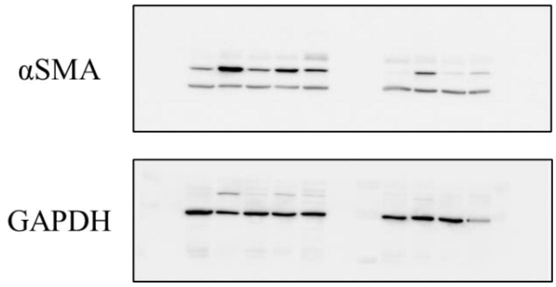

Supplement: Supplementary file 1 [file ijms-22-04178-s001.zip › ijms-1177695-suppl-resubmitted.pdf]
